# Supplementary material for: Key role of eg* band broadening in nickel-based oxyhydroxides on coupled oxygen evolution mechanism
Source: Nat Commun. 2023 Nov 18;14:7488. doi: 10.1038/s41467-023-43302-2 (PMC10657368; doi:10.1038/s41467-023-43302-2)
Supplement: Supplementary file 1 — Supplementary information [file 41467_2023_43302_MOESM1_ESM.pdf]

# Supplementary information

## Key role of $e_g^*$ band broadening in nickel-based oxyhydroxides on coupled oxygen evolution mechanism


Haoyin Zhong[1], Qi Zhang[1], Junchen Yu[1], Xin Zhang[1], Chao Wu[2,3], Hang An[1], Yifan Ma[1], Hao Wang[1], Jun Zhang[1], Yong-Wei Zhang[4], Caozheng Diao[5], Zhi Gen Yu[4*], Shibo Xi[2*], Xiaopeng Wang[1,3*], Junmin Xue[1*]

**Affiliations**

[1]Department of Materials Science and Engineering, National University of Singapore, Singapore, 117575.

[2] Institute of Sustainability for Chemical, Energy and Environment (ISCE[2]), Agency for Science, Technology and Research, Singapore, 627833.

[3] College of Materials Science and Engineering, Sichuan University, Chengdu, China, 610065.

[4]Institute of High Performance Computing, Agency for Science, Technology and Research, Singapore, 138632, Singapore

[5]Singapore Synchrotron Light Sources (SSLS), National University of Singapore, 117603, Singapore

*Corresponding author. Email: msexuejm@nus.edu.sg (Junmin Xue); xi_shibo@isce2.a-star.edu.sg (Shibo Xi); msewxia@nus.edu.sg (Xiaopeng Wang); yuzg@ihpc.a-star.edu.sg (Zhigen Yu)

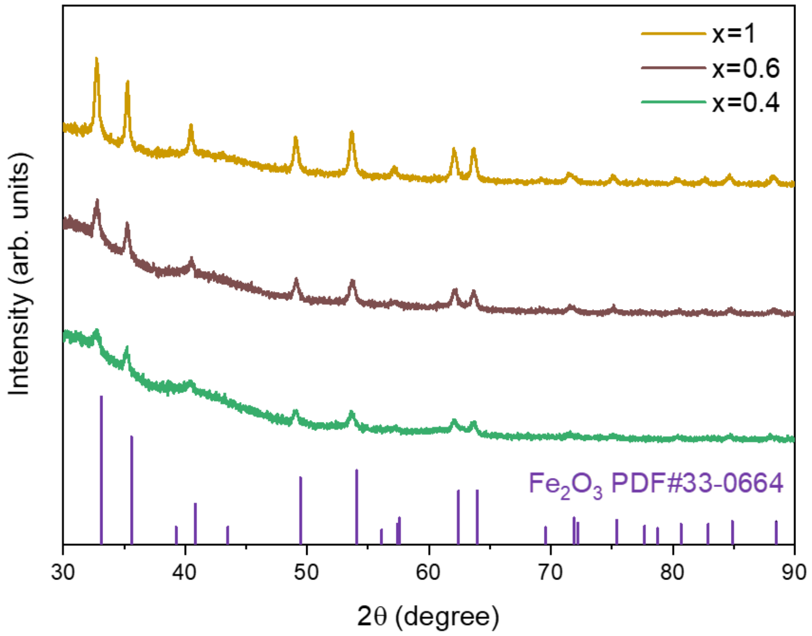


**Supplementary Fig. 1.** XRD patterns of hydrothermal derived samples with ratio of Ni : Fe = (1-x ) : x, where x=0.4,0.6, 1.

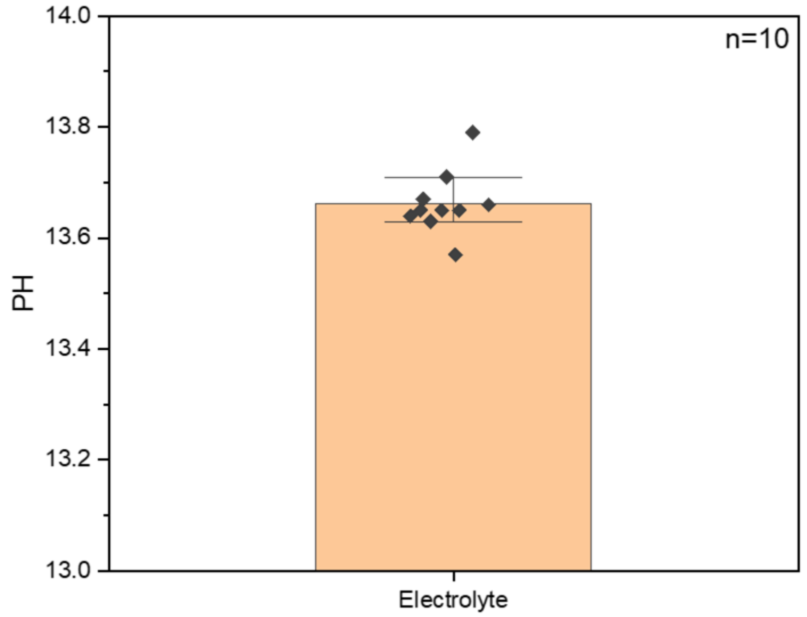


**Supplementary Fig. 2.** PH value of the purified 1M KOH and the error bars represent mean ± standard error.

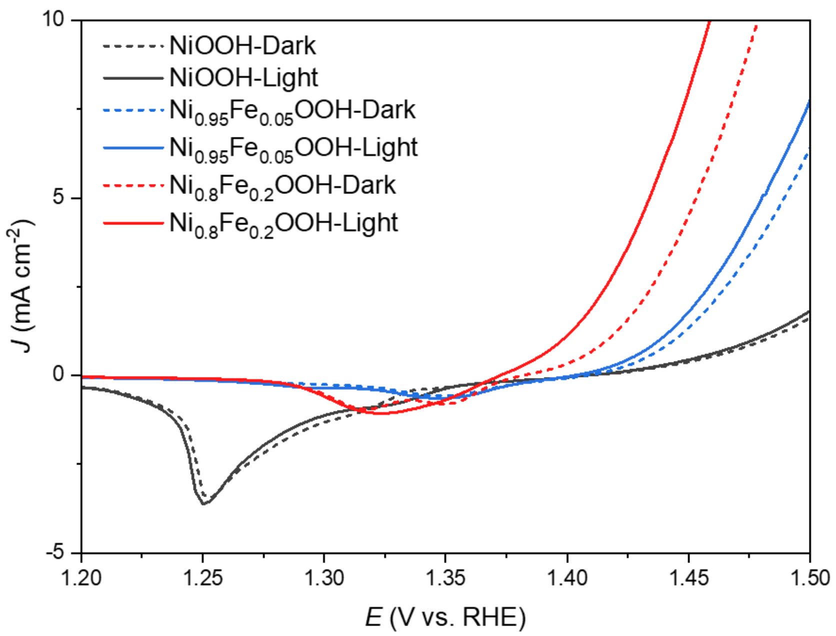


**Supplementary Fig. 3.** Enlarged LSV polarization curves of $Ni_{1-x}Fe_xOOH$ (x = 0, 0.05, 0,2) from Fig.1a based on a backward scan conducted at a scan rate of 0.1 mV $s^{-1}$.

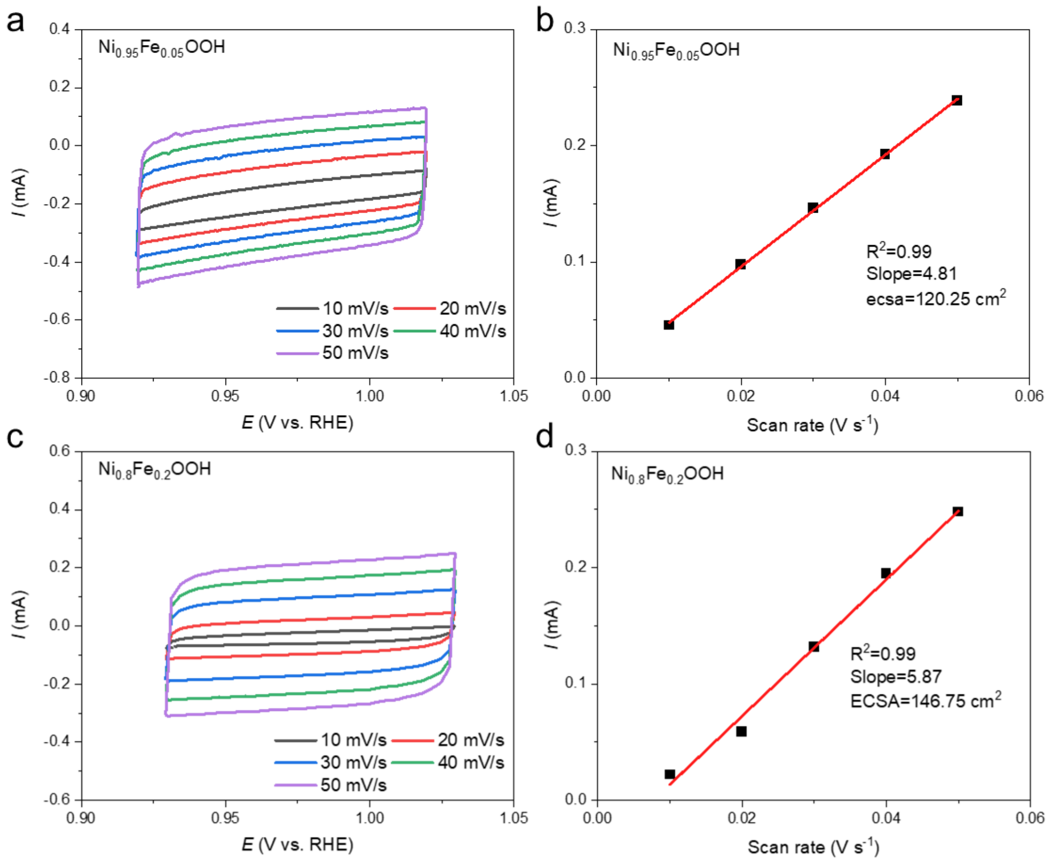


**Supplementary Fig. 4. ECSA characterization.** The Cyclic voltammograms curves and current (*I*) vs. scan rate plots of the (a)-(b) $Ni_{0.95}Fe_{0.05}OOH$ (c)- (d) $Ni_{0.8}Fe_{0.2}OOH$. Plots of current vs. scan rate data were obtained from CV curves at 0.98 V. The ECSA for all samples was calculated as: $Ni_{0.95}Fe_{0.05}OOH$ (120.25 $cm^2$), $Ni_{0.8}Fe_{0.2}OOH$ (146.75 $cm^2$).

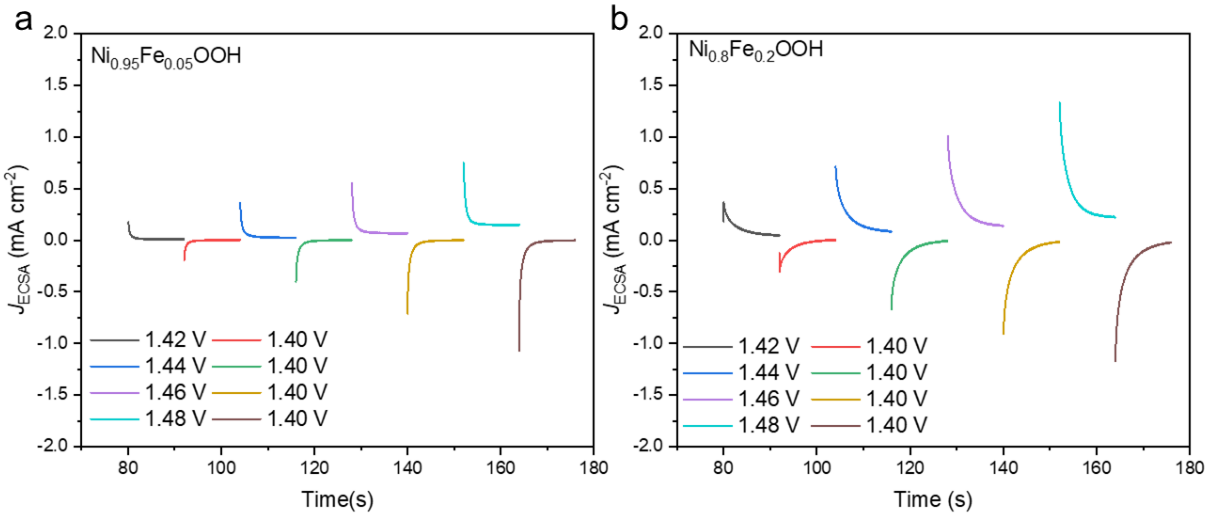


**Supplementary Fig. 5. Pulse pulse-voltammetry measurements** (a) $Ni_{0.95}Fe_{0.05}OOH$. (b) $Ni_{0.8}Fe_{0.2}OOH$

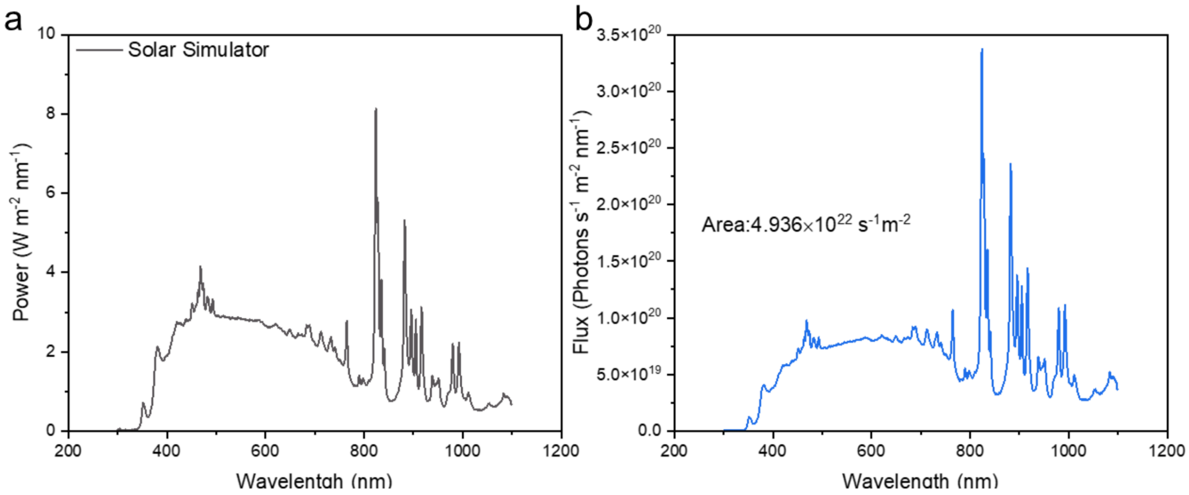


**Supplementary Fig. 6. Theoretical maximum photon flux calculation.** (a) Incident power from the sun. AM 1.5 G. (b) The corresponding photon flux for the AM 1.5 G tilt solar flux spectrum, the integrated region of the solar spectrum represents the theoretical maximum number of photons ($4.936\times10^{22}$ $m^{-2}$ $s^{-1}$) that can be collected.

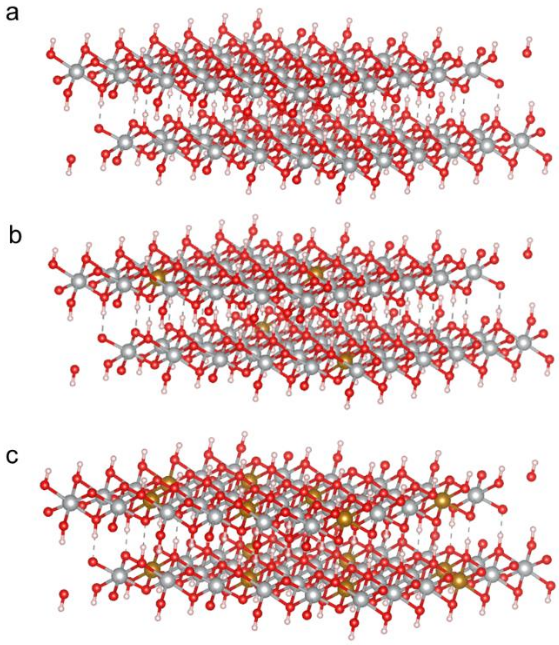


**Supplementary Fig. 7. Simulation models** (a) NiOOH. (b) $Ni_{0.95}Fe_{0.05}OOH$. (c) $Ni_{0.8}Fe_{0.2}OOH$. The optimized unit cell of layered NiOOH was expanded to 6×3 supercells (a=17.556 Å and b=17.739 Å) containing 72 Ni, 72 H and 144 O atoms with a vacuum thickness of 18 Å for Fe embedding systems. The simulation results for $Ni_{0.95}Fe_{0.05}OOH$ and $Ni_{0.8}Fe_{0.2}OOH$ are corresponded to computational models of $Fe_4Ni_{68}$ and $Fe_{14}Ni_{58}$, respectively.

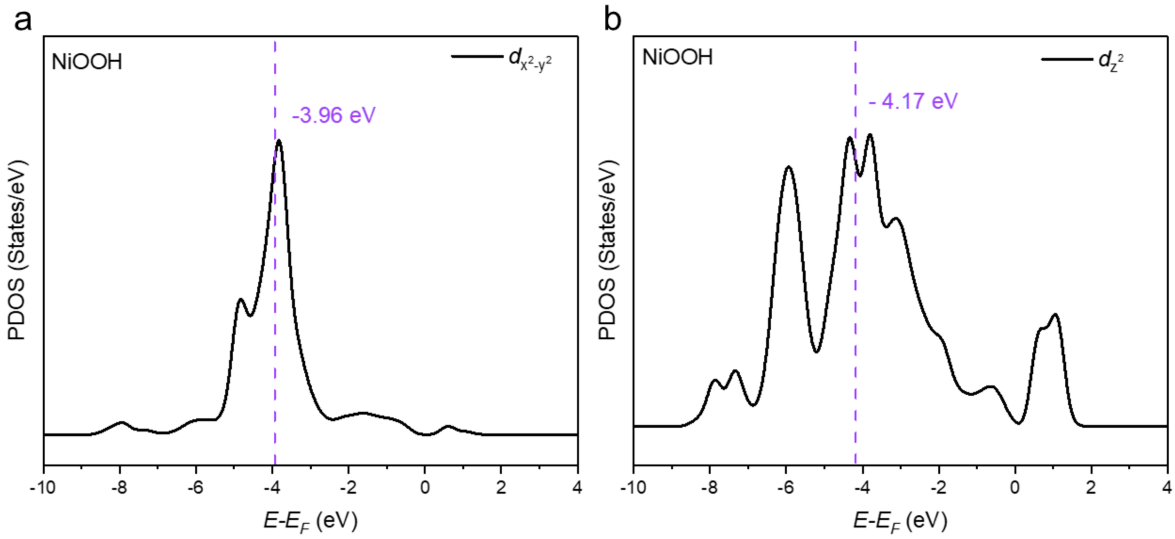


**Supplementary Fig. 8. Band center of NiOOH** (a) $d_{x^2-y^2}$ orbital. (b) $d_{z^2}$ orbital.

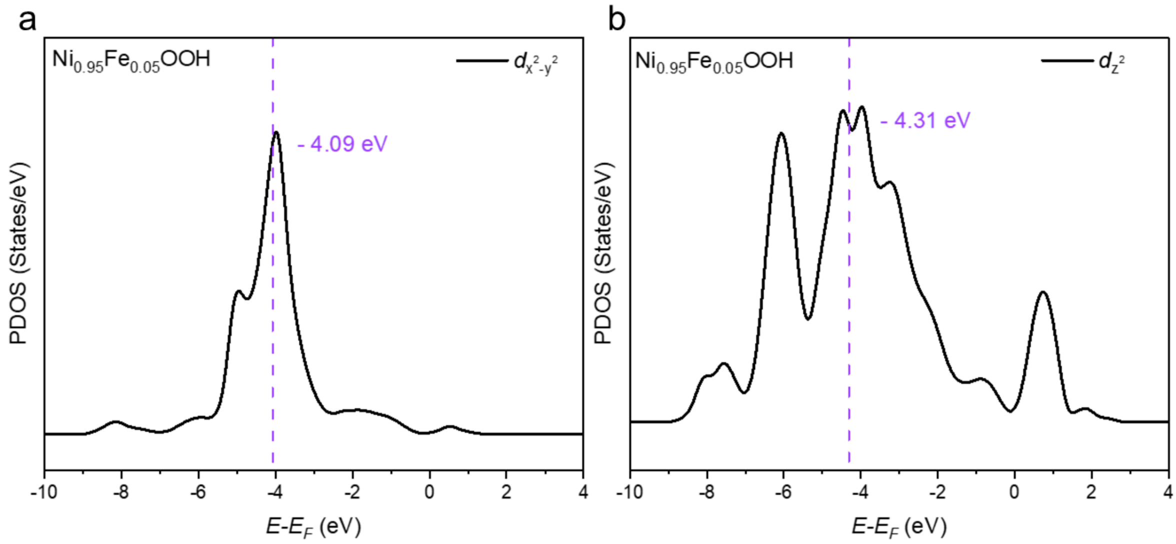


**Supplementary Fig. 9. Band center of $Ni_{0.95}Fe_{0.05}OOH$** (a) $d_{x^2-y^2}$ orbital. (b) $d_{z^2}$ orbital.

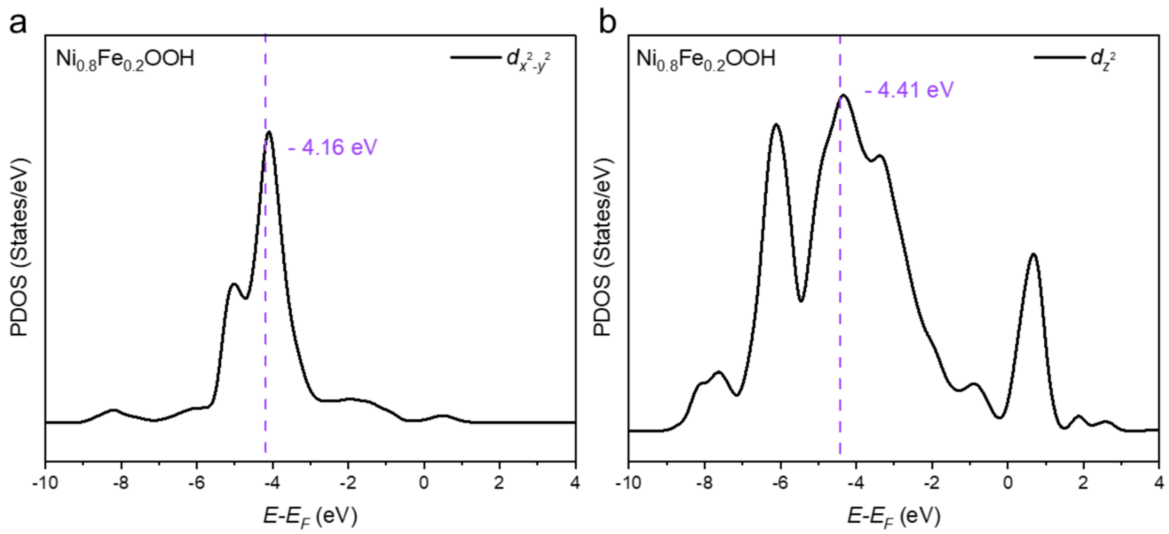


**Supplementary Fig. 10. Band center of $Ni_{0.8}Fe_{0.2}OOH$** (a) $d_{x^2-y^2}$ orbital. (b) $d_{z^2}$ orbital.

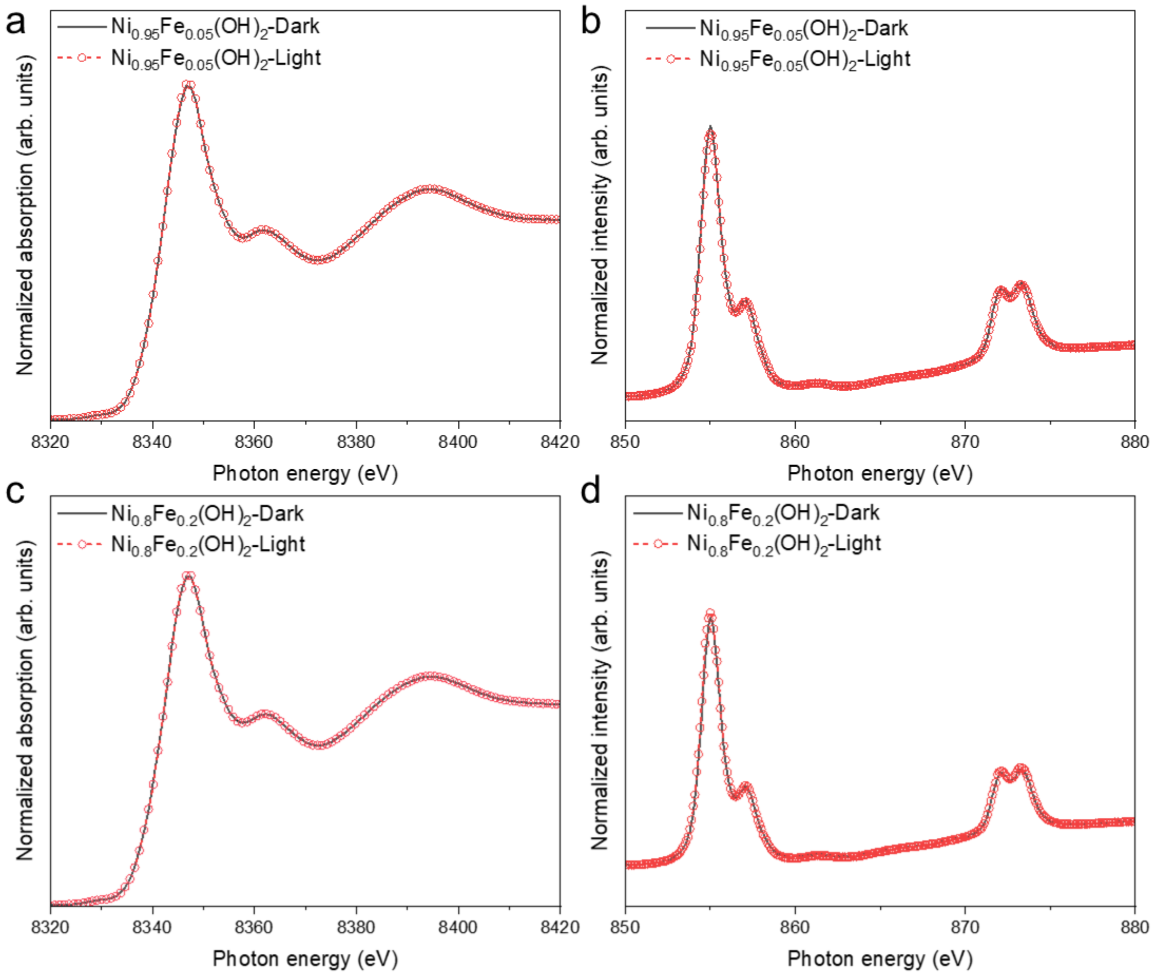


**Supplementary Fig. 11. Structural comparison before OER and after OER.** Ni *K*-edge spectra (a) and *L*-edge spectra (b) of $Ni_{0.95}Fe_{0.05}(OH)_2$ after OER under dark or light condition; Ni *K*-edge spectra (c) and *L*-edge spectra (d) of $Ni_{0.8}Fe_{0.2}(OH)_2$ after OER under dark or light condition. Here, it should be noted that $Ni_{0.95}Fe_{0.05}OOH$ and $Ni_{0.8}Fe_{0.2}OOH$ samples were completely reduced to $Ni_{0.95}Fe_{0.05}(OH)_2$ and $Ni_{0.8}Fe_{0.2}(OH)_2$ using ethanol[1] to avoid the possible effects on the XAS results caused by the self-discharge of $Ni^{3+}$.

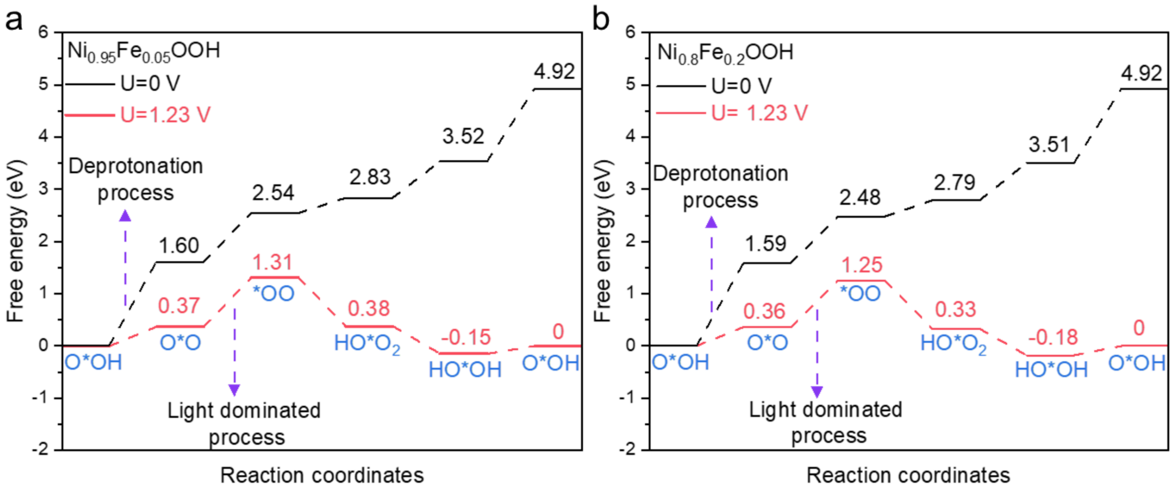


**Supplementary Fig. 12. Calculated reaction free energies following the COM route** (a) $Ni_{0.95}Fe_{0.05}OOH$. (b) $Ni_{0.8}Fe_{0.2}OOH$.

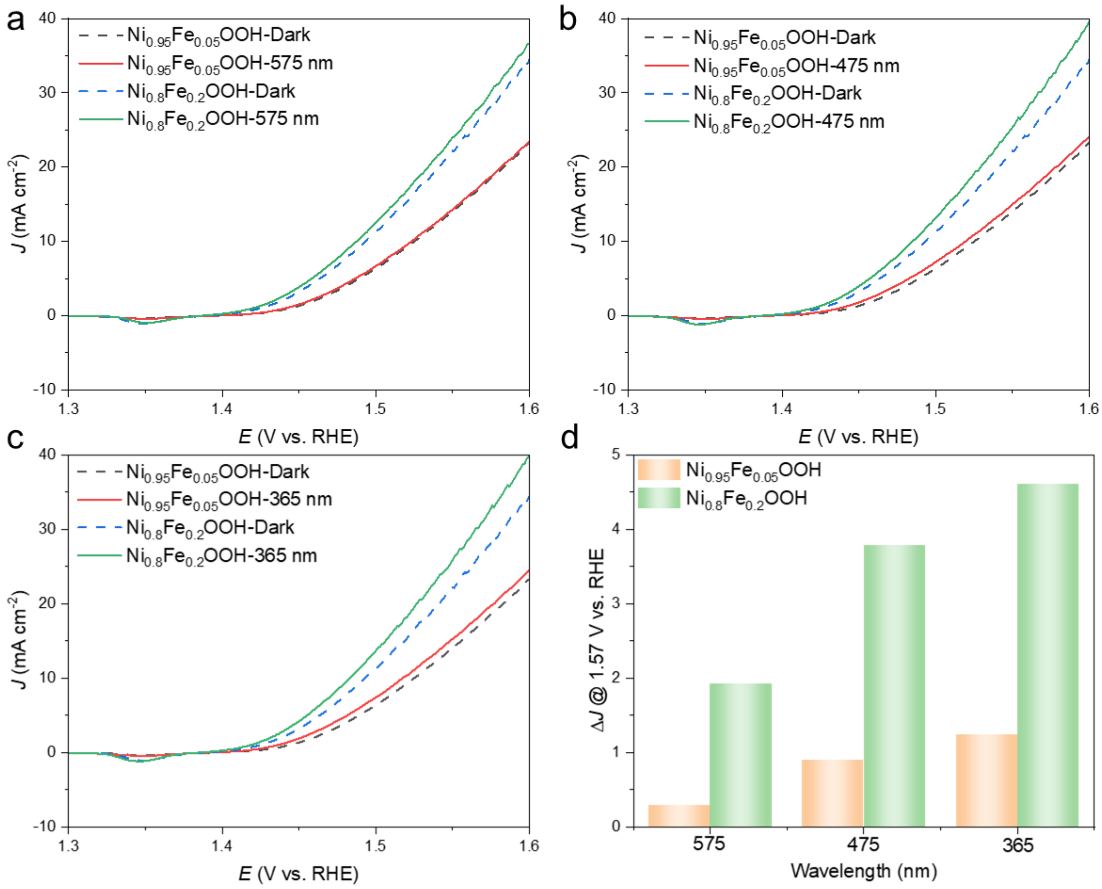


**Supplementary Fig. 13. LSV polarization curves under light with different wavelength** (a) LSV polarization curves of $Ni_{0.95}Fe_{0.05}OOH$ and $Ni_{0.8}Fe_{0.2}OOH$ under dark and light with wavelength at 575 nm; (b) LSV polarization curves of $Ni_{0.95}Fe_{0.05}OOH$ and $Ni_{0.8}Fe_{0.2}OOH$ under dark and light with wavelength at 475 nm; (c) LSV polarization curves of $Ni_{0.95}Fe_{0.05}OOH$ and $Ni_{0.8}Fe_{0.2}OOH$ under dark and light with wavelength at 365 nm; (d) Comparison of the current density at 1.57 V vs. RHE between dark condition and light condition at different wavelength (575nm, 475nm, 365nm) for $Ni_{1-x}Fe_xOOH$ (x = 0.05, 0.2).

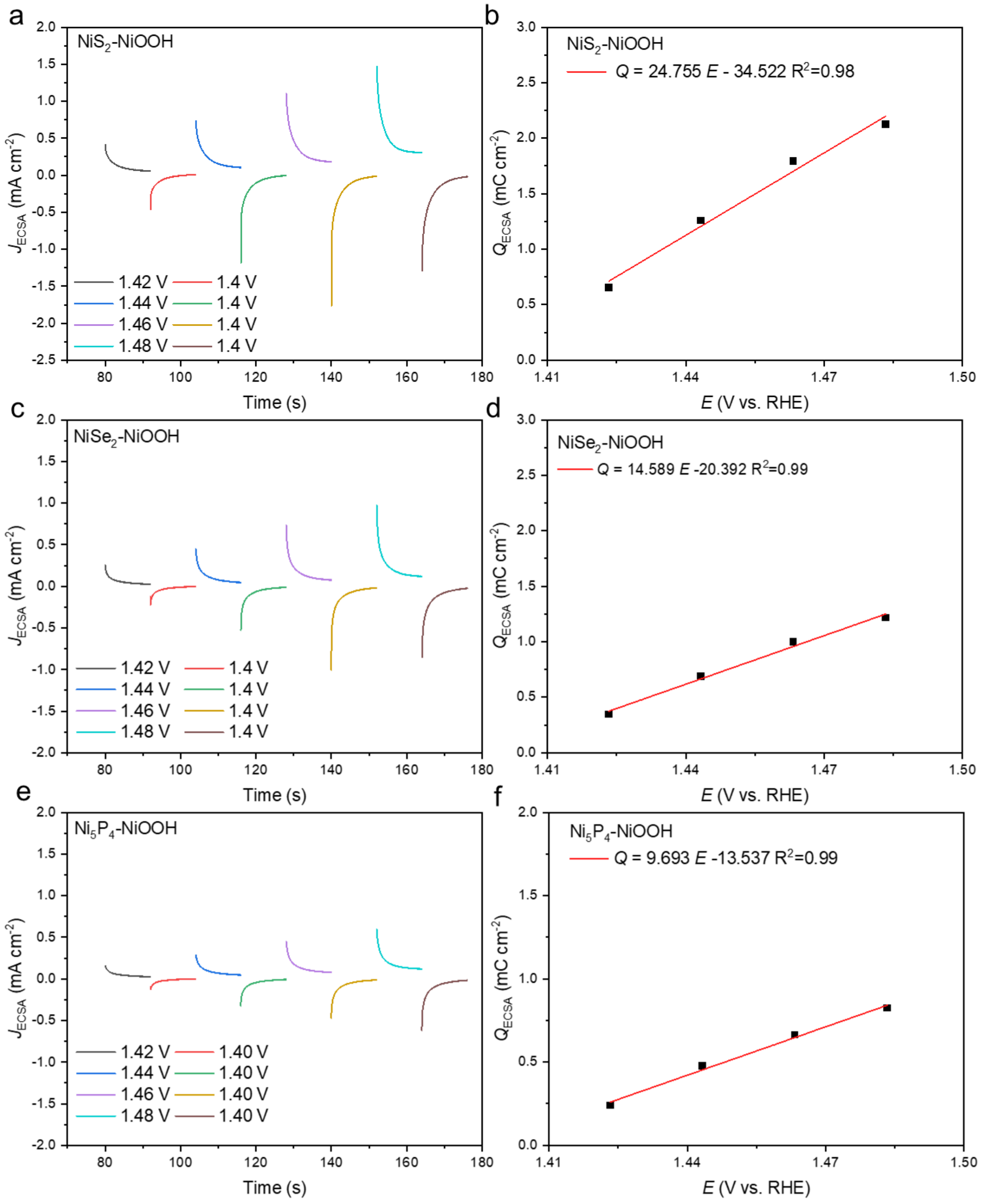


**Supplementary Fig. 14. Pulse-voltammetry measurements and the integrated charge versus potential** (a) (b) $NiS_2$-NiOOH. (c) (d) $NiSe_2$-NiOOH. (e) (f) $Ni_5P_4$-NiOOH.

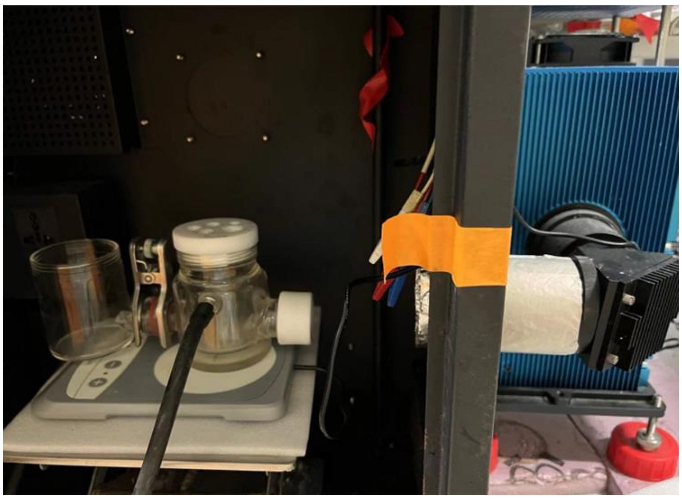

**Supplementary Fig. 15.** The experimental set-up for LSV measurements under dark and light conditions

## Supplementary References

1 Sun, S., Zhou, Y., Hu, B., Zhang, Q. & Xu, Z. J. Ethylene glycol and ethanol oxidation on spinel Ni-Co oxides in alkaline. *Journal of The Electrochemical Society* **163**, H99 (2015).
