# Supplementary material for: Key role of eg* band broadening in nickel-based oxyhydroxides on coupled oxygen evolution mechanism
Source: Nat Commun. 2023 Nov 18;14:7488. doi: 10.1038/s41467-023-43302-2 (PMC10657368; doi:10.1038/s41467-023-43302-2)
Supplement: Supplementary file 3 — Description of Additional Supplementary Files [file 41467_2023_43302_MOESM3_ESM.pdf]

Description of Additional Supplementary Files

Supplementary Data 1: CONTCAR of the optimized $Ni_{1-x}Fe_xOOH$ (x = 0, 0.05, 0.2) models
